# Improving Romantic Relationship Functioning Among Young Men With First-Episode Psychosis: Impact of a Novel Group Intervention

**DOI:** 10.1177/01454455231186586

**Published:** 2023-07-26

**Authors:** Briana Cloutier, Tania Lecomte, Felix Diotte, Justin Lamontagne, Amal Abdel-Baki, Jean-Gabriel Daneault, Marie Eve Gélineau Rabbath, Alexandre de Connor, Cécile Perrine

**Affiliations:** 1 Université de Montréal, QC, Canada; 2 Centre hospitalier de l’Université de Montréal, Clinique JAP, QC, Canada; 3 Services ambulatoires de santé mentale adulte 2ième ligne, Montréal, QC, Canada; 4 Centre hospitalier universitaire de Montpellier, Centre de rétablissement et de réhabilitation Jean-Minvieille, Montpellier, France; 5 Établissement public de santé mentale Caen, Unité de réhabilitation psychosociale Ariane, Caen, France

**Keywords:** romantic relationships, sexuality, psychosis, group intervention, videoconferencing

## Abstract

Previous research has highlighted many of the challenges faced by individuals with psychosis in romantic relationships. The present study aimed to evaluate the impact of a novel group intervention for men with first-episode psychosis (FEP) on dating success, romantic and sexual functioning, self-esteem, self-stigma, mentalizing skills, and symptomatology, while using a repeated single-case experimental design and comparing results across two treatment modalities (i.e., in-person or online). Twenty-seven participants from five treatment sites completed a 12-week group intervention. Qualitative data was also collected to assess participants’ subjective experiences with the program. In both modalities, significant improvements were observed for romantic functioning, mentalizing skills, and symptomatology, with effect sizes ranging from small to large. Several participants also attended more dates and entered committed relationships after the intervention. Most participants were satisfied with the program and many felt that they had learned new skills and gained confidence in dating. Future research should replicate these findings in larger and more inclusive samples.

## Introduction

The ability to form and maintain healthy romantic relationships is considered a critical developmental task and defining feature of the transition to adolescence or early adulthood (Collins, 2003; Furman et al., 2007). Failure to initiate and sustain positive romantic experiences during this period can have deleterious consequences for an individual’s long-term well-being and broader social functioning, as limited learning experiences hinder interpersonal skills acquisition (Rauer et al., 2013; Schulenberg et al., 2004). Unfortunately, emerging psychiatric symptoms may also interfere with one’s achievement of important social milestones, such as developing romantic relationships (Goldstein et al., 2017; Redmond et al., 2010). This would seem to be especially true for individuals living with a psychotic disorder, for whom romantic relationships are much more difficult to navigate (Redmond et al., 2010).

Previous research has identified several obstacles to successful romantic relationships among individuals with psychotic disorders, including stigma (de Jager & McCann, 2017; Latour-Desjardins et al., 2019; Van Dorn et al., 2005) and attachment difficulties (Gabinio et al., 2018; Harder, 2014; Pillay et al., 2018), social-cognitive deficits (Fett et al., 2011; Sprong et al., 2007), and medication side effects (e.g., sexual dysfunctions and weight gain; Addington et al., 2003; de Boer et al., 2015). Indeed, traumatic and discriminatory experiences are frequently reported (Van Dorn et al., 2005; Varese et al., 2012), as are low self-esteem and poor communication skills (Dickinson et al., 2007; Lysaker et al., 2007). While both men and women with a psychotic disorder are more likely to face additional challenges when dating compared to members of the general population, men may be at an even greater disadvantage due to an earlier and often more severe onset of the first psychotic episode (Cotton et al., 2009; Thorup et al., 2007).

In addition to pharmacological treatments, numerous psychosocial interventions have been developed to facilitate recovery from psychosis. Cognitive-behavioral therapy (CBT) has been found to reduce positive symptoms (e.g., hallucinations and delusions; Lecomte et al., 2019; Turner et al., 2014), whereas social-skills training (SST) has been shown to improve negative symptoms (e.g., apathy and social withdrawal; Kurtz & Mueser, 2008; Turner et al., 2014). An intervention aiming to improve romantic relationship functioning among individuals with psychosis could incorporate principles from both CBT and SST in order to target previously identified issues in dating and relationships. For example, cognitive restructuring might be employed to address negative or distressing beliefs that contribute to avoidance or conflict in romantic relationships (Gould et al., 2001). At the same time, various interpersonal skills (e.g., using verbal and non-verbal cues to signal one’s interest, communicating emotions and problem-solving with a partner) can also be modeled by therapists and practiced by clients until sufficient competence is achieved (Browne et al., 2020). Thus, a program combining evidenced-based techniques from each approach may be more likely to promote success in romantic and sexual encounters than either treatment model alone.

Group therapy has been recognized as an effective treatment modality for a wide variety of psychological conditions (Burlingame et al., 2003; Burlingame et al., 2016). In psychiatry, group interventions may even be favored over individual therapy because of their cost-effectiveness (Tucker & Oei, 2007) and distinct therapeutic processes (e.g., normalization of experiences, vicarious learning) that can only occur in a group setting (Yalom & Leszcz, 2005). Among individuals with psychosis specifically, group therapy has been found to promote better clinical, social, and functional outcomes (Burlingame et al., 2020; Orfanos et al., 2015), particularly at earlier stages of the disorder (i.e., first-episode psychosis (FEP); Lecomte et al., 2008; Lecomte et al., 2012).

The COVID-19 pandemic has generated significant interest in the use of videoconferencing to conduct therapy. Several studies have found videoconferencing to be a feasible and acceptable method for delivering psychological interventions (Backhaus et al., 2012; Batastini et al., 2020), although most research to date has focused on individual therapy. A recent study on group teletherapy for people with early psychosis suggests that this treatment modality may produce similar results to traditional in-person group interventions (Lecomte et al., 2020). Given the increasing use of videoconferencing in therapy and other benefits of this treatment modality (e.g., greater accessibility to services in remote areas), further research on group teletherapy is needed (Lecomte et al., 2020).

In light of the unique difficulties faced by individuals with psychotic disorders with respect to their romantic relationships and repeated requests for greater assistance in this area (Cloutier et al., 2021), our team developed a novel group intervention for men with psychosis seeking to improve their dating lives. Having obtained preliminary evidence of the program’s acceptability and feasibility in a recent pilot study (Hache-Labelle et al., 2020), the goal of the present study was to extend these findings by evaluating its effect on dating success, romantic and sexual functioning, self-esteem and self-stigma, mentalizing skills, and symptomatology in a different sample of young men with FEP using a within-subject, repeated single-case experimental design, as well as compare results across two treatment modalities (i.e., in-person or online via videoconferencing). Qualitative data was also collected to assess participants’ subjective experience with the program.

The primary dependent variables of this study were dating success, romantic functioning, and sexual functioning. These outcomes were specifically selected because they directly reflected the objectives of the proposed intervention. Secondary dependent variables (self-esteem, self-stigma, mentalizing skills, symptomatology) were also included due to their indirect but nonetheless meaningful relationship to dating and sexuality. When compared to baseline, it was hypothesized that:

1. Dating success (i.e., number of dates and number of committed relationships) would increase after receiving the intervention.
2. Romantic and sexual functioning would increase after receiving the intervention.
3. Self-esteem and mentalizing skills would increase after receiving the intervention.
4. Self-stigma and symptomatology would decrease after receiving the intervention.

## Methods

### Design

This mixed-method study used a multi-site, repeated single-case experimental design (SCED), with both primary and secondary outcome measures, as well as two different treatment modalities (in-person and online via videoconferencing). SCEDs aim to test the effect of an intervention within subjects, by using a small number of participants and repeated, systematic measurements (Krasny-Pacini & Evans, 2017). Participants act as their own controls over time as baseline phases are compared to subsequent phases where a treatment is generally introduced and removed (Smith, 2012). Assignment to treatment modality was non-randomized as the intervention was offered in the format that the treatment site had pre-determined. Thus, some treatment sites only offered the intervention in-person, while others only offered it online. These decisions were largely influenced by COVID-19 restrictions at the time of the study.

### Participants

A total of 27 men between 20 and 36 years of age were recruited by service providers from three outpatient clinics in Montreal, Canada, as well as two outpatient clinics in Montpellier and Caen, France. Participants who expressed interest in the study were referred to the research coordinator. A poster advertising the project and the research team’s contact information was also added to the waiting rooms of the clinics. To be eligible for the program, participants had to identify as a heterosexual cisgender male, have experienced at least one psychotic episode (affective or non-affective), have stabilized symptoms (i.e., using medication for at least two months), and be able to read and understand French. Restrictions on gender identity and sexual orientation were included because we aimed for a homogenous sample, which is generally recommended when evaluating a novel treatment with a small sample size (Jager et al., 2017). The presence of a psychotic episode or psychotic disorder diagnosis was confirmed by participants’ treatment teams and consultation of their medical files. Individuals were excluded from the study if they were unable to consent to the study, if they had an organic disorder or mental disability, and if they were currently receiving psychotherapy services focusing on relational skills. Descriptive statistics for the study sample can be found in Table 1.

**Table 1.** Participant Baseline Characteristics.

Characteristic	In-person group (*n* = 14)	Online group (*n* = 13)	Total (*N* = 27)
Age	28.15 ± 4.82	25.69 ± 5.22	26.96 ± 5.07
Recruited in Montreal	5 (35.7%)	13 (100%)	18 (66.7%)
Education level			
No high school diploma	5 (35.7%)	6 (46.2%)	11 (40.7%)
High school diploma	3 (21.4%)	4 (30.8%)	7 (25.9%)
College degree	1 (7.1%)	1 (7.7%)	2 (7.4%)
Bachelor’s degree	3 (21.4%)	2 (15.4%)	5 (18.5%)
Master’s or Doctorate degree	2 (14.3%)	0 (0%)	2 (7.4%)
Occupation			
Working	1 (7.1%)	4 (30.8%)	5 (18.5%)
Studying	2 (14.3%)	6 (46.2%)	8 (29.6%)
Supported employment or vocational program	7 (50.05%)	1 (7.7%)	8 (29.6%)
No occupation	2 (14.3%)	2 (15.4%)	4 (14.8%)
Other (e.g., volunteering)	2 (14.3%)	0 (0%)	2 (7.4%)
Living situation			
Alone	7 (50.0%)	1 (7.7%)	8 (29.6%)
With family	6 (42.9%)	11 (84.6%)	17 (63.0%)
Residential center	1 (7.1%)	1 (7.7%)	2 (7.4%)
Primary diagnosis			
Schizophrenia-spectrum disorder	11 (78.6%)	6 (46.2%)	17 (63.0%)
Mood disorder with psychotic features	1 (7.1%)	3 (23.1%)	4 (14.8%)
Other psychosis (e.g., episode)	2 (14.3%)	4 (30.8%)	6 (22.2%)

### Measures

Participants were asked to fill out a brief socio-demographic questionnaire at baseline which included items relating to age, gender, education level, civil and work status, as well as participants’ current financial and living situation, and psychiatric diagnoses. All other measures were administered at each of the four assessment sessions (T1 to T4).

Romantic functioning was measured using three instruments: the Romantic Relationship Functioning Scale (RRFS; Bonfils et al., 2016), the First Episode Social Functioning Scale—Intimacy subscale (FESFS; Lecomte et al., 2014), and a brief descriptive questionnaire on dating and committed relationships. The RRFS contains 22 items and 3 subscales (Risks, Resources, and Stigma). These subscales measure respondents’ subjective perceptions of possible risks, personal resources, and stigmatizing experiences in dating and romantic relationships. Higher subscale and global scores indicate better romantic relationship functioning (Risks and Stigma scores are reverse-coded). Sample items for the RRFS include “*I am good at communicating in romantic relationships*” and “*I am scared that a romantic partner might take advantage of me*.” The FESFS Intimacy subscale contains 11 items and asks about attitudes and behaviors relating to recent dating experiences, romantic and sexual partners, and emotional intimacy. Higher global scores indicate better intimate relationship functioning. Sample items for the FESFS Intimacy subscale include “*I feel I am able to share feelings, inner thoughts, and be close with my stable boy/girlfriend or spouse (when I have one)*” (attitude) and “*In the last three months, I have had sexual relations with someone*” (behavior). The descriptive questionnaire on dating and committed relationships was created for the purpose of the study and asked two questions: how many dates they had attended in the last month and whether they had entered a committed relationship in the last month (defined as both partners agreeing to exclusivity). If participants indicated having recently begun a committed relationship, they were also asked how long they had been dating this person (number of weeks).

Sexual functioning was measured using the Multidimensional Sexuality Questionnaire (MSQ; Snell et al., 1993), which contains 60 items and examines several tendencies associated with human sexuality, including awareness about sexual needs and preferences, emotions associated with sexual experiences, and sexual satisfaction. These tendencies are grouped into two categories reflecting positive and negative sexual functioning, respectively. Higher positive sexual functioning scores indicate between better sexual functioning, while higher negative sexual functioning scores indicate poorer sexual functioning. Sample items for the MSQ include “*I am very alert to changes in my sexual desires*” and “*I sometimes am fearful of sexual activity*.” The RRFS, the FESFS, and the MSQ have demonstrated adequate reliability and validity in prior studies (Bonfils et al., 2016; Lecomte et al., 2014; Snell et al., 1993).

Self-esteem was measured using the Self-Esteem Rating Scale – Short Form (SERS-SF; Lecomte et al., 2006), which contains 20 assessing positive self-esteem (e.g., belief that one is interesting or has a good sense of humor) and negative self-esteem (e.g., feelings of shame or inferiority). Higher, positively-valued global scores indicate better self-esteem. Sample items for the SERS-SF include “*I feel that I make a good impression on others*” and “*I feel that I am likely to fail at things I do*.” Self-stigma was measured using the Internalized Stigma of Mental Illness Scale (ISMIS; Ritsher et al., 2003), which contains 29 items and examines stigmatizing views including alienation and stereotype endorsement. Higher global scores indicate greater self-stigma. Sample items for the ISMIS include “*People with mental illness cannot live a good, rewarding life*” and “*I am disappointed in myself for having a mental illness*.” Social cognition abilities, specifically mentalization skills, were assessed using the Stories Test - Abridged Version (Achim et al., 2012). Respondents were asked to read 10 short stories and answer questions about the presence and nature of any implicit meanings in each story. Higher global scores on the Stories Test indicate better mentalizing skills. The SERS-SF, the ISMIS, and the Stories Test have also demonstrated adequate psychometric properties (Achim et al., 2012; Lecomte et al., 2006; Ritsher et al., 2003). Finally, a short qualitative questionnaire assessing participants’ subjective experience with the program was also completed at T3. Respondents were asked by a research assistant what they liked and disliked about the group intervention, as well as whether they were generally satisfied with the program.

Symptomatology was measured using the Brief Psychiatric Rating Scale—Expanded Version (BPRS-E; Lukoff et al., 1986), a semi-structured interview conducted by raters trained to the UCLA gold standard (i.e., high inter-rater reliability with expert scores). The BPRS-E contains 26 items evaluating various aspects of psychopathology (e.g., anxiety, depression, suicidal ideation, delusions, hallucinations, etc.). Interviewers asked initial probe and follow-up questions before rating each symptom domain. Higher subscale and global scores indicate more frequent and severe symptoms. Sample items for the BPRS-E include “*Have you felt sad, depressed or down in the past two weeks*?” and “*Do you ever hear voices or sounds no one else hears*?” The BPRS-E has demonstrated good psychometric properties in psychiatric samples (Mouaffak et al., 2010).

### Procedure

The project was evaluated and approved by the Centre hospitalier de l’Université de Montréal’s ethics committee and was funded by the Fonds de Recherche du Québec and the Centre de recherche interdisciplinaire sur les problèmes conjugaux et les agressions sexuelles. This study followed a multi-site, repeated SCED. Although randomized controlled trials (RCTs) are considered the gold standard for evaluating the efficacy of a treatment, an SCED was favored in the present study because (1) drop-out rates tend to be extremely high (up to 80%) among individuals with a serious mental illness when they are randomized to a wait-list or treatment-as usual condition, which compromises internal validity, and (2) there is currently no validated group treatment for romantic relationships that could be used as an active control condition (Lecomte et al., 2012). According to Smith (2012), SCEDs must show replication effects across a minimum of three conditions (e.g., subjects, settings, behaviors) to be of satisfactory methodological quality. Consequentially, different groups of participants received our intervention at different locations, and treatment effects were evaluated for different outcomes.

The outcomes of interest for this study included dating success, romantic and sexual functioning, self-esteem and self-stigma, mentalizing skills, and symptomatology. Given prior research showing improvements on both positive and negative symptoms of psychosis with CBT and SST interventions (Turner et al., 2014), we expected to see a decrease in participants’ symptoms after our program. We also expected to see improvements on participants’ romantic and sexual functioning due to our intervention’s emphasis on dating and romantic skills, as well as advice for addressing sexual questions and concerns. Self-esteem was also expected to increase after the intervention as a consequence of increased knowledge acquisition and associated self-confidence. Similarly, self-stigma was expected to decrease after the intervention as a result of participants’ enhanced intimacy skills. Finally, we expected to see improvements on participants’ mentalizing skills due to the program’s teaching and practicing of the CBT model (i.e., seeking alternative explanations) in order to decipher ambiguous social situations. Each of these outcomes were assessed by a trained research assistant 4 weeks prior to the beginning of the intervention (T1), once immediately before (T2) and once immediately after the intervention (T3), as well as 4 weeks following the end of the intervention (T4). SCED methodologies must include more than one baseline assessment as well as more than one post-therapy follow-up assessment. As such, we included two pre-treatment assessments in order to demonstrate no change or difference on our outcome variables before the intervention was introduced, as well as two post-treatment assessments to demonstrate maintenance of treatment effects. Assessments were either conducted in-person or online via Zoom, according to participants’ preferences and research assistants’ availabilities. The intervention itself occurred over a total of 12 weeks, with each session lasting 90 minutes and occurring once per week. Participants received a stipend (60$) for completing the questionnaires and interviews.

### Treatment

The intervention, called *Power of Two*, is a group therapy program that combines CBT and SST principles and is designed to help individuals establish and maintain romantic relationships. It covers a wide range of topics, such as how to meet someone and show interest, how to recognize one’s own and others’ thoughts and feelings, sexual expectations, and how to manage conflicts and solve problems effectively (see Table 2 for a description of each session). The content and format of the intervention is based on insights gained from prior studies with the population of interest (e.g., McCann, 2010; Redmond et al., 2010), as well as previous research conducted by our team (Latour-Desjardins et al., 2019; Pillay et al., 2018). The treatment manual was also modified based on comments from clinicians, sex and couples therapists, and people with lived experience. Two mental health professionals affiliated with each participating clinic delivered the intervention to groups containing between four and eight participants. Although fidelity to the manual was not explicitly evaluated, therapists were required to complete a 4-hour intensive training session with the program’s creator (T.L.). Clinical supervision was also offered on an as-needed basis.

**Table 2.** Program Description.

Session title	Content
Session 1: Am I ready?	Rules and goals of the group, personal reasons for wanting to date, pros and cons of dating (support vs stress).
Session 2: Dating–Part 1	How and where to meet people, pros and cons of each method, how to describe oneself to potential partners.
Session 3: Dating–Part 2	How to get ready for a date, how to show interest or recognize it in someone else, small talk, reciprocal conversation (role-play).
Session 4: From dating to going out	What am I looking for, my values, how do I know if the person is right for me?
Session 5: My qualities as a partner and disclosure about mental illness	What are my qualities, what can I offer, when should I (if ever) disclose my mental health status and how? Pros and cons of each scenario.
Session 6: Recognizing my feelings and sharing them	How do I recognize when I am feeling specific emotions, how do I know if I’m in love, how can I share positive and negative feelings, how do I cope with difficult emotions?
Session 7: What is going on?	How to inquire about what the other person is thinking? Review of CBT model with alternative explanations and fact-checking.
Session 8: My story and my fears	What scares me about being in a relationship (abandonment, dependency, loss of independence)? How to talk about my fears, how to find the right distance between myself and the other person?
Session 9: Sex and intimacy—Part 1	Expectations. When should sex be proposed? How is consent determined? Pornography vs reality. Sexual preferences, exploration, identity.
Session 10: Sex and intimacy—Part 2	Protection/contraception. Sexual problems–What to do?
Session 11: Managing conflicts	Problem-solving steps and strategies.
Session 12: Communication and happiness	Communication skills during conflicts. Strategies to keep a relationship healthy and happy. Review of the group module.

### Analyses

Descriptive analyses were conducted for data on attendance, as well as participants’ dating success. Mixed ANOVAs and post-hoc paired samples t-tests with Bonferroni adjustments were computed to evaluate changes on each scale over time, while also controlling for age, treatment site, education level, and psychiatric diagnosis. This analytical strategy was chosen because it allowed us to compare differences in scores across four timepoints (within-subjects factor), in addition to testing for interaction effects between time and treatment modality (between-subjects factor). Effect sizes were calculated using Cohen’s *d* (Cohen, 1988). Qualitative data was examined and summarized by two of the authors, J.G.D. and M.E.G.R., using a frequency count strategy. Discrepancies were resolved through revision of key words and phrases until consensus was reached.

## Results

### Quantitative Data

A total of seven groups were offered during the course of the project, with three groups being offered in-person and four groups being offered online. Two participants were excluded from our analyses due to low attendance (i.e., present for less than 6/12 sessions). Only participants who completed at least one pre- and one post-evaluation were retained for the analyses. In all, 22 completed all four evaluations, while five completed three evaluations. For the 27 participants included in our analyses, average attendance was 9.44 out of 12 sessions.

**Figure 1.**
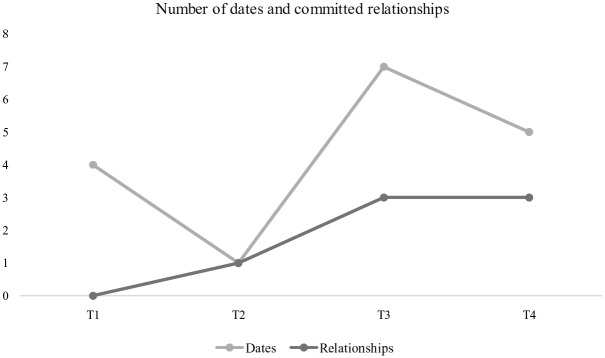
Trends in dating and committed relationships across time.

At T1 (baseline), none of the participants were in a committed relationship and only 4 (14.8%) had recently been on a date. At T2 (pre-group), one participant (3.7%) indicated being in a committed relationship, while another participant had recently gone on a date. At T3 (post-group), three participants were in a committed relationship (11.1%) and seven other participants had recently been on a date (25.9%). At T4 (4-week follow-up), three participants were still in committed relationships, while five other participants (18.5%) had recently gone on dates. These trends are presented graphically in Figure 1.

Within-group analyses revealed improvements on several outcomes. As can be seen in Table 3, romantic functioning (RRFS-Total) was significantly higher after the intervention. This change was associated with a medium effect size (Cohen’s *d* = 0.65). Age was also found to influence romantic functioning, with younger participants displaying significantly greater romantic relationship functioning than older participants, *F*(1, 18.84) = 12.50, *p* < .01. Both treatment delivery options produced similar results on this outcome, as revealed by the non-significant Time x Modality interaction term. To better understand observed changes in romantic functioning, RRFS subscale scores were also analyzed. Scores on the Resources subscale (RRFS-Resources) and Stigma subscale (RRFS-Stigma) significantly increased following the intervention. Effect sizes were large (Cohen’s *d* = 1.22) and small (Cohen’s *d* = 0.23) for these two outcomes, respectively. The Time x Modality interaction terms were also non-significant. No changes were observed on the Risks subscale (RRFS-Risks).

**Table 3.** Results of Main Analyses.

Outcomes	Mean and standard error (*SE*)								*p*-Value	Timepoint comparisons						Cohen’s *d* T1–T4	Time *x* modality interaction
	Time 1		Time 2		Time 3		Time 4			T4-T1	T3-T1	T2-T1	T4-T2	T3-T2	T4-T3		
	Mean	*SE*	Mean	*SE*	Mean	*SE*	Mean	*SE*		*p*-Value	*p*-Value	*p*-Value	*p*-Value	*p*-Value	*p*-Value		*p*-Value
RRFS-Total	2.96	0.11	3.11	0.10	3.23	0.10	3.31	0.10	**.019**	**.013**	**.043**	.450	.139	.432	1.00	0.65	.491
RRFS-Resources	2.75	0.17	2.87	0.15	3.19	0.16	3.22	0.15	**<.001**	**<.001**	**<.001**	.621	**<.001**	**<.001**	1.00	1.22	.177
RRFS-Stigma	3.07	0.19	3.29	0.20	3.22	0.17	3.52	0.17	**.022**	.097	1.00	.780	1.00	1.00	.088	0.23	.799
RRFS-Risks	3.08	0.18	3.16	0.15	3.27	0.15	3.22	0.15	.551	1.00	1.00	1.00	1.00	1.00	1.00	0.27	.272
FESFS-Attitudes	3.00	0.11	3.01	0.12	3.09	0.10	3.06	0.11	.663	1.00	1.00	1.00	1.00	1.00	1.00	0.21	.915
FESFS-Behavior	1.48	0.09	1.46	0.09	1.80	0,17	1.79	0.15	**.034**	.163	.215	1.00	.069	.075	1.00	0.51	.980
MSQ-Positive	2.78	0.14	2.95	0.16	3.06	0.15	2.94	0.15	.072	1.00	.079	.384	1.00	1.00	.909	0.39	.949
MSQ-Negative	2,38	0,18	2,35	0,19	2,20	0,17	2,18	0,17	.526	1.00	1.00	1.00	1.00	1.00	1.00	0.26	.080
SERS-Positive	42.78	2.77	42.90	2.54	45.32	2.74	43.37	2.37	.297	1.00	1.00	1.00	1.00	.554	1.00	0.27	.088
SERS-Negative	−32.21	2.76	−32.24	3.27	−31.85	3.29	−31.35	3.20	.952	1.00	1.00	1.00	1.00	1.00	1.00	0.09	.781
ISMI-Total	1.85	0.12	1.79	0.12	1.73	0.14	1.69	0.12	.312	.369	.841	1.00	1.00	1.00	1.00	0.39	.358
ST-Mentalization	16.54	0.97	17.49	1.04	18.92	1.08	19.65	1.04	**.003**	**.002**	**.010**	.523	**.024**	.082	1.00	0.86	.553
BPRS-Total	42.39	1.96	41.50	2.27	36.42	1.58	35.29	1.52	**<.001**	**<.001**	**<.001**	1.00	**.002**	**.002**	.933	1.08	.665
BPRS-Negative	9.49	0.54	9.14	0.52	7.67	0.41	7.21	0.33	**<.001**	**<.001**	**.006**	1.00	**.001**	**.005**	.745	0.87	.932

*Note*. Numbers in bold are statistically significant at *p* < .05.

Intimacy behaviors (FESFS-B) were also significantly higher after the intervention, with a medium effect size (Cohen’s *d* = 0.51). The Time × Modality interaction term was non-significant, indicating that results were similar across treatment delivery options. No changes were observed for the Intimacy attitudes (FESFS-A) subscale. Mentalizing skills (ST-Mentalization) also significantly increased following the intervention. The effect size of this change was large (Cohen’s *d* = 0.86) and the Time × Modality interaction term was non-significant.

Although changes were non-significant, positive sexual functioning (MSQ-Positive) scores increased after the intervention. Given the small-to-medium effect size (Cohen’s *d* = 0.39) associated with this change, a significant effect may have been observed with a larger sample size. The Time x Modality interaction term was non-significant but trending for negative sexual functioning (MSQ-Negative). As with positive sexual functioning, a significant effect may have been detected with a larger sample. Self-esteem (SERS-Positive and SERS-Negative) and self-stigma (ISMI-Total) scores did not improve significantly over time.

Global symptomatology (BPRS-Total) was significantly reduced after the intervention. The effect size associated with this change was large (Cohen’s *d* = 1.08). Treatment site was also found to influence global symptomatology, with participants at one site displaying significantly less psychiatric symptoms than participants at each of the four other sites, *F*(4, 22.36) = 7.20, *p* < .01. Given the program’s emphasis on social skills training, negative symptomatology (BPRS-Negative) was also examined and found to be significantly lower after the intervention. The effect size was large for this outcome (Cohen’s *d* = 0.87). The Time × Modality interaction terms were non-significant for both BPRS outcomes.

### Qualitative Data

At the end of the program, the participants were asked what they liked about the modules, what they disliked and whether they were satisfied with the program. Of all the participants, 74% (21 out of 27) gave their opinion.

In general, participants liked having the opportunity to talk, exchange, and socialize, in a friendly, safe, and judgement-free space. Many also appreciated receiving concrete tools. For example, four participants particularly appreciated receiving ideas of activities they could do to meet potential partners. Others liked to reflect on relational issues. For example, three participants mentioned enjoying discussions on conflict resolution. In addition, two participants indicated that they enjoyed learning about women’s point of view of love.

What may have been disliked by one participant was, simultaneously, appreciated by another. One participant felt that there lacked practice exercises, more precisely, role-playing, while two other participants found role-playing to be excessive. A few participants had trouble sharing about their sexuality. Two participants were bothered by psychotic symptoms present in other group members. Another participant said there lacked sharing and participation from other participants. One participant was disappointed at the end of the program because he did not find a romantic partner. Only one participant mentioned not having appreciated the videoconferencing platform.

The majority of participants (81% or 17 out of 21) said they were satisfied with the modules. Five of these underlined having learnt new things and three mentioned having gained confidence due to improved skills in communication and conflict resolution. Some participants did not elaborate on what satisfied them. A few mentioned dissatisfaction including feeling judged (one participant), feeling inadequately informed (one participant) and feeling general discomfort (one participant). Other participants suggested adding individual sessions in order to address matters specific to them and expressed interest towards mixed-gender groups.

## Discussion

This study aimed to evaluate the impact of a novel group intervention for young men with FEP. We also sought to compare results across two treatment modalities—in-person or online. Similar improvements were observed on multiple outcomes for both modalities. For instance, romantic functioning was increased significantly after the intervention for participants in each delivery condition. This change was primarily driven by improvements in participants’ Resources scores, suggesting that they left feeling more prepared for dating. This is unsurprising given the content of the program (e.g., discussions on fun and inexpensive date ideas, advice for interpreting romantic signals). Stigma scores were also improved after the intervention, though to a lesser degree. Different ways of addressing one’s mental health history with a potential romantic partner were presented during the intervention, which may have helped reduce participants’ concerns about experiencing discrimination while dating. Interestingly, younger participants displayed significantly greater romantic functioning than older participants. This difference could potentially be explained by older participants’ greater exposure to previous negative romantic experiences and, consequentially, less self-confidence and more fears surrounding dating. Future research should investigate how age influences dating for adults with psychosis, as well as its implications for treatment.

Intimacy behaviors also increased significantly after the intervention for participants in each delivery condition, indicating that participants were engaging more with potential romantic partners following therapy. This was also corroborated by descriptive data on dating outcomes, which showed an increase in the number of dates and committed relationships after the intervention. However, participants’ attitudes towards intimacy did not improve over time. One possible explanation for this finding is that new experiences may be needed in order to change attitudes about intimacy (Frye et al., 2012; Glasman & Albarracin, 2006), as many individuals with psychosis have had few or mostly negative prior dating experiences (Latour-Desjardins et al., 2019). This may also be true for self-esteem and self-stigma, two outcomes that did not improve after the intervention. Thus, a greater focus on previous traumatic experiences may be needed to help shift long-standing unfavorable perceptions about oneself and other people in the context dating (de Jager et al., 2021; White et al., 2021). Our findings suggest that stigmatizing attitudes could potentially be improved with our intervention (small effect size for RRFS-Stigma), but it would be interesting to evaluate whether adjunct trauma-based interventions could enhance these effects, particularly among individuals with a greater history of trauma exposure. Similarly, while our small sample size prevents us from drawing any firm conclusions, improvements in sexual functioning may have been observed if more of the program’s content had been more devoted to sexuality, as only two sessions focused on this topic. Additional support for complex sexuality concerns may needed as part of an integrated care package for certain participants.

In addition to dating success and romantic functioning, mentalizing skills also increased significantly after the intervention for participants in each delivery condition. This was expected due to the program’s emphasis on understanding emotions and implicit messages through numerous discussions and role-plays. Indeed, many participants expressed that this part of the program was especially helpful to them, as it offered concrete information on how to respond more effectively in realistic dating situations. Finally, both global and negative symptoms decreased significantly after the intervention for participants in each delivery condition. Although this cannot be verified in the present study, it is likely that interacting regularly with other people as part of a group promotes symptomatic remission, even if this occurs at a distance via videoconferencing (Lecomte et al., 2015; Lecomte et al., 2020). Participants at one treatment site also differed significantly from other treatment sites with respect to global symptomatology. This variation could be explained by baseline differences in symptoms, as participants at this treatment site were considerably less symptomatic before starting therapy than those at the other treatment sites.

These findings should be considered in light of the present study’s limitations. Most importantly, a small sample size may have contributed to power-related issues, restricting our ability to understand the treatment’s impact on certain outcome variables. A larger study with more participants is needed to replicate these findings. In future studies, a two-armed RCT (*Power of Two* versus existing CBT or SST intervention) or a three-armed RCT (*Power of Two* versus a manualized CBT or SST intervention versus treatment-as-usual (TAU)) would allow us to further reduce the risk of bias as well as evaluate whether our intervention is superior to standard care and, to some extent, a similar, established intervention. However, SCEDs can provide strong evidence for an intervention’s efficacy through triangulation (i.e., replication of effects across a minimum of three conditions), which was demonstrated in the current study (Smith, 2012). Although an RCT design comparing the current intervention to a similar, evidence-based intervention (e.g., SST or CBT program for psychosis) would have strengthened our findings, we are unaware of any existing active comparison that specifically targets romantic and/or sexual functioning. Another important limitation is the sociocultural context in which the study was conducted. It is impossible to assess exactly how the COVID-19 pandemic might have influenced the study’s findings, although our results are largely consistent with those of the pilot study that was conducted before the pandemic (Hache-Labelle et al., 2020). Finally, the majority of our results were based on self-reported data. However, two outcomes of interest, mentalizing skills and symptomatology, were evaluated by trained raters. The fact that significant improvements were observed across different assessment measures, in addition to different treatment sites, increases confidence in our findings (Smith, 2012).

In conclusion, this study was the first to evaluate the impact of a novel group intervention focusing on romantic relationships in young men with FEP and offered in two treatment modalities (in-person versus online). Results suggest that this program led to improvements in participants’ dating success, romantic functioning, mentalizing skills, and symptomatology, with both traditional and videoconferencing treatment modalities being equally effective. Group members were largely satisfied with the content of the sessions and indicated benefitting from the intervention. In an effort to increase accessibility, future studies should replicate these findings in larger, more inclusive samples (e.g., including individuals with different gender and sexual orientation identities, other age groups, other languages). Furthermore, future research should investigate which components of the program are most effective, as well as which individuals might respond better or more poorly to the intervention (de Villiers et al., 2018; Hofmann & Hayes, 2019). Targeting trauma outside of group therapy, either before or during the intervention, may also be necessary for some participants to better benefit from this treatment (de Jager et al., 2021; White et al., 2021).
